# Evaluating the Effect of Lenvatinib on Sorafenib-Resistant Hepatocellular Carcinoma Cells

**DOI:** 10.3390/ijms222313071

**Published:** 2021-12-02

**Authors:** Tingting Shi, Hisakazu Iwama, Koji Fujita, Hideki Kobara, Noriko Nishiyama, Shintaro Fujihara, Yasuhiro Goda, Hirohito Yoneyama, Asahiro Morishita, Joji Tani, Mari Yamada, Mai Nakahara, Kei Takuma, Tsutomu Masaki

**Affiliations:** 1 Department of Gastroenterology and Neurology, Faculty of Medicine, Kagawa University, 1750-1 Ikenobe, Miki 761-0793, Japan; 92m7v9@med.kagawa-u.ac.jp (K.F.); kobara@med.kagawa-u.ac.jp (H.K.); n-nori@med.kagawa-u.ac.jp (N.N.); joshin@med.kagawa-u.ac.jp (S.F.); goda0717@med.kagawa-u.ac.jp (Y.G.); hyoneyam@med.kagawa-u.ac.jp (H.Y.); asahiro@med.kagawa-u.ac.jp (A.M.); georget@med.kagawa-u.ac.jp (J.T.); mari-yamada@med.kagawa-u.ac.jp (M.Y.); m-nakahara@med.kagawa-u.ac.jp (M.N.); k-takuma@med.kagawa-u.ac.jp (K.T.); 2 Life Science Research Center, Kagawa University, 1750-1 Ikenobe, Miki 761-0793, Japan; iwama@med.kagawa-u.ac.jp

**Keywords:** lenvatinib, sorafenib-resistant, hepatocellular carcinoma, FGFR4, autophagy, microRNA

## Abstract

Hepatocellular carcinoma (HCC) is one of the major causes of cancer-related deaths worldwide. Sorafenib has been used as a first-line systemic treatment for over a decade. However, resistance to sorafenib limits patient response and presents a major hurdle during HCC treatment. Lenvatinib has been approved as a first-line systemic treatment for advanced HCC and is the first agent to achieve non-inferiority against sorafenib. Therefore, in the present study, we evaluated the inhibition efficacy of lenvatinib in sorafenib-resistant HCC cells. Only a few studies have been conducted on this topic. Two human HCC cell lines, Huh-7 and Hep-3B, were used to establish sorafenib resistance, and in vitro and in vivo studies were employed. Lenvatinib suppressed sorafenib-resistant HCC cell proliferation mainly by inducing G1 cell cycle arrest through ERK signaling. Hep-3B sorafenib-resistant cells showed partial cross-resistance to lenvatinib, possibly due to the contribution of poor autophagic responsiveness. Overall, the findings suggest that the underlying mechanism of lenvatinib in overcoming sorafenib resistance in HCC involves FGFR4-ERK signaling. Lenvatinib may be a suitable second-line therapy for unresectable HCC patients who have developed sorafenib resistance and express FGFR4.

## 1. Introduction

Hepatocellular carcinoma (HCC) is the most common primary liver cancer, with an increasing incidence over the past few decades in various populations; it is one of the major causes of cancer-related deaths worldwide [1,2]. Only a small fraction of patients is diagnosed with early stages of the disease, when curative strategies—liver resection (LR), ablative techniques, and orthotopic liver transplantation (OLT)—can be employed [3]. For patients with advanced HCC, systemic therapy is available, with prolonged overall survival (OS) rates. 

Sorafenib, an oral multi-kinase inhibitor approved in 2008, targets the Raf–MEK–ERK pathway and several receptor tyrosine kinases, including vascular endothelial growth factor receptors (VEGFRs) 2 and 3, platelet-derived growth factor receptor (PDGFR), FMS-related tyrosine kinase 3 (FLT3), Ret, and c-Kit [4]. The median survival time with sorafenib, used as a first-line systemic therapy for the past decade, was nearly 3 months longer than that with the placebo (10.7 months vs. 7.9 months; hazard ratio (HR) 0.69; *p* < 0.001) [4]. However, intrinsic and acquired resistance to sorafenib remains a huge challenge, with only approximately 30% of patients responsive to sorafenib [4,5]. The primary resistance of HCC cells to sorafenib is postulated to be associated with genetic heterogeneity—the overexpression of epidermal growth factor receptor (EGFR) or ligand may lead to sustained activation of EGFR downstream signaling and drug resistance to sorafenib [6,7]. Acquired resistance to sorafenib, which often develops within six months [5], may be associated with several factors, such as the phosphatidylinositol 3-kinase (PI3K)/Akt pathway, autophagy, epithelial-mesenchymal transition (EMT), tumor microenvironment, epigenetic regulation, microRNAs (miRNAs), and “vessel co-option”—the ability of tumors to hijack the existing vasculature in organs such as the lungs or liver, thereby limiting the need for angiogenesis [7,8]. 

Second- and later-line systemic treatments are needed for patients who fail to respond or are intolerant to sorafenib. The multitargeted multi-kinase inhibitors regorafenib [9] and cabozantinib [10] were approved in 2017 and 2018, respectively, as second-line drugs. Further, PD-1 immune checkpoint inhibitors, nivolumab and pembrolizumab, have been approved as second-line therapies for patients with advanced HCC [11].

Lenvatinib is a multi-kinase inhibitor that targets VEGFR 1–3, fibroblast growth factor (FGF) receptors 1–4, PDGFR-α, RET, and KIT [12]. A recent clinical trial showed that the median survival time of 13.6 months with lenvatinib (95% CI 12.1–14.9) was non-inferior to that with sorafenib (12.3 months, 10.4–13.9; HR 0.92, 95% CI 0.79–1.06) in untreated advanced HCC [13]. Thus, lenvatinib has been approved as a first-line systemic treatment for unresectable advanced HCC. Further, some evidence indicates that lenvatinib may be used as a second-line treatment for patients who are intolerant to sorafenib or following sorafenib failure [14,15,16]. 

To explore the possible advantage of lenvatinib and the underlying mechanisms in overcoming sorafenib-resistance in HCC we developed sorafenib-resistant cell lines and performed in vitro and in vivo experiments; further, we identify the expression of miRNAs associated with the effect of lenvatinib. Only a few studies have been conducted on this topic.

## 2. Results

### 2.1. Lenvatinib Inhibits Sorafenib-Resistant HCC Cell Proliferation 

**Figure 1:**
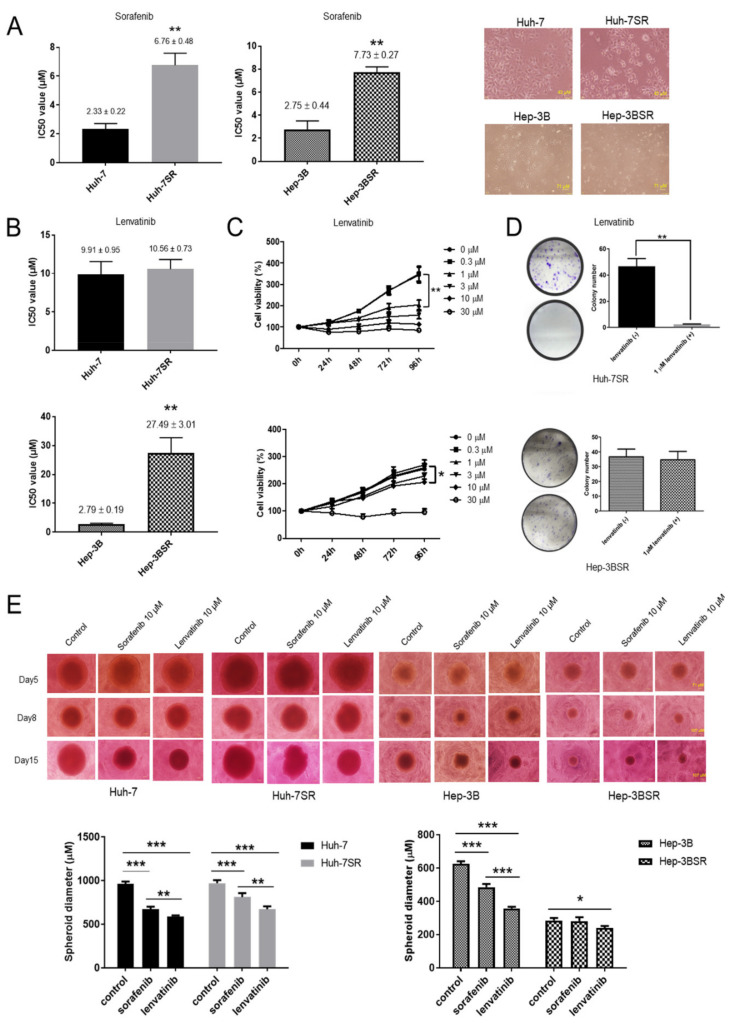
Lenvatinib inhibits the proliferation of sorafenib-resistant human hepatocellular carcinoma (HCC) cells. (**A**) Cytotoxic effect of sorafenib (IC_50_): Cells were treated for 48 h with increasing concentrations of sorafenib, and dimethyl sulfoxide (DMSO) was used as a negative control. IC_50_ of Huh-7 sorafenib-resistant (Huh-7SR) cells was 2.9-fold that of Huh-7 cells, and IC_50_ of Hep-3BSR cells was 2.81-fold that of Hep-3B cells. (**B**) Cytotoxic effect of lenvatinib (IC_50_): Cells were treated for 48 h with increasing concentrations of lenvatinib, and dimethyl sulfoxide (DMSO) was used as a negative control. Lenvatinib IC_50_ values of Huh-7 and Huh-7SR cells were not significantly different. Lenvatinib IC_50_ of Hep-3B sorafenib-resistant (Hep-3BSR) cells was 9.85-fold that of Hep-3B cells. (**C**) Anti-proliferative effect of lenvatinib measured using cell viability assay: Huh-7SR and Hep-3BSR cells were treated for 96 h with the indicated concentrations of lenvatinib or DMSO. The relative cell number was normalized with the control. (**D**) After 1 μM lenvatinib or DMSO treatment, the colonies formed were fixed and stained with crystal violet. (**E**) Three-dimensional tumor spheroid assay was performed to evaluate the effect of lenvatinib and sorafenib on the wild-type and sorafenib-resistant HCC cell proliferative ability. The representative images of spheroids are shown (scale bar: 5th day, 71 μm; 8th and 15th days, 107 μm). Spheroid diameters were measured on 15th day. Data are represented as means ± standard error of mean (SEM) or means ± standard deviation (SD) of at least three independent experiments. * *p* < 0.05, ** *p* < 0.01, *** *p* < 0.001.

The IC_50_ of Huh-7SR cells (6.76 ± 0.48 μM) was 2.9-fold higher than that of Huh-7 cells (2.33 ± 0.22 μM), and the IC_50_ of Hep-3BSR cells (7.73 ± 0.27 μM) was 2.81-fold higher than that of Hep-3B cells (2.75 ± 0.44 μM) when exposed to sorafenib (Figure 1A). The lenvatinib IC_50_ values were not significantly different between Huh-7 (9.91 ± 0.95 μM) and Huh-7SR (10.56 ± 0.73 μM) cells; however, the lenvatinib IC_50_ of Hep-3BSR cells (27.49 ± 3.01 μM) was 9.85-fold that of Hep-3B cells (2.79 ± 0.19 μM) (Figure 1B). The Huh-7SR and Hep-3BSR cells were treated with 0.3, 1, 3, 10, and 30 μM lenvatinib for 96 h, and the anti-proliferative effect of lenvatinib was assessed using the cell viability assay. DMSO-treated cells were used as controls. Lenvatinib inhibited cell proliferation in the Huh-7SR and Hep-3BSR cells in a dose- and time-dependent manner. Further, 1 μM lenvatinib showed a significant effect in the Huh-7SR cells; however, 10 μM lenvatinib was required in the Hep-3BSR cells (Figure 1C). In the colony formation assay, during a long culture period (14 days), a small dose (1 μM) of lenvatinib showed an obvious anti-proliferative effect in the Huh-7SR cells, but not in the Hep-3BSR cells (Figure 1D). This may be due to the partial cross-resistance of Hep-3BSR cells to lenvatinib; the anti-proliferative effect of lenvatinib in Hep-3BSR cells needs a higher dose than Huh-7SR cells. To further verify the anti-proliferative effect of lenvatinib in Huh-7SR and Hep-3BSR cells, a 3D tumor spheroid assay was performed. An in vitro dosage (10 μM) of sorafenib and lenvatinib was used in the 3D spheroid culture of both wild-type and resistant cells; this showed that lenvatinib (10 μM) has an advantageous anti-proliferative effect in all cells (Huh-7, Huh-7SR, Hep-3B, and Hep-3BSR) compared with sorafenib (10 μM). Moreover, sorafenib could partially inhibit Huh-7SR cell proliferation but not in Hep-3BSR cells (Figure 1E). 

### 2.2. Lenvatinib Induces Apoptosis and Cell Cycle G1 Phase Arrest, Decreases Invasion and Migration Ability, and Regulates the Expression of Angiogenesis-Related Proteins in Huh-7SR Cells

**Figure 2:**
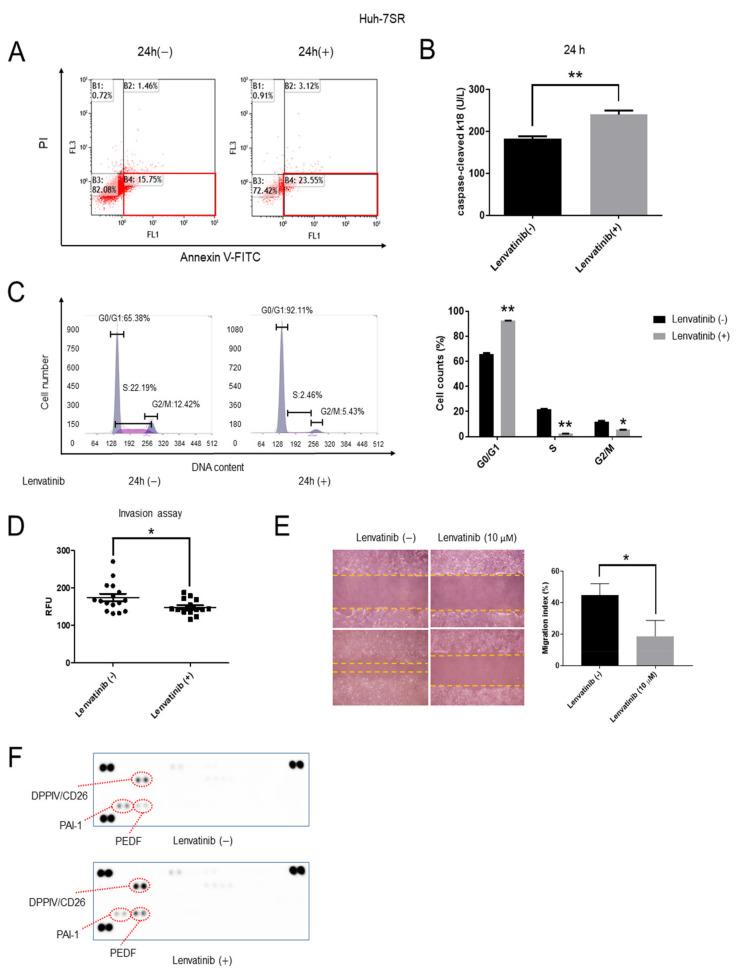
In Huh-7SR cells, lenvatinib induces apoptosis and cell cycle G1 phase arrest, reduces invasion and migration, and modulates the expression of proteins associated with angiogenesis. (**A**) Huh-7SR cells were treated with 10 μM lenvatinib or dimethyl sulfoxide (DMSO) for 24 h, and the level of apoptosis was measured by staining with annexin V and propidium iodide (PI) using flow cytometry. Lenvatinib treatment increased the proportion of early apoptotic cells in the Huh-7SR population. Lower right square represents early apoptosis. (**B**) The expression of caspase-cleaved cytokeratin 18 (cCK-18) was determined using Enzyme Linked Immunosorbent Assay (ELISA) after 24 h of treatment with 10 μM lenvatinib. (**C**) Huh-7SR cells treated with 10 μM lenvatinib or DMSO were analyzed using flow cytometry to determine the number of cells in each phase of the cell cycle (left panel). Representative cell cycle histograms are presented (right panel). Lenvatinib blocked the cell cycle at the G1 phase. (**D**) Invasion ability of lenvatinib-treated Huh-7SR cells was decreased. (**E**) Wound-healing assay comparing the motility of Huh-7SR cells treated with lenvatinib or DMSO. The wound-healing area was analyzed using the ImageJ software. (**F**) Representative expression of angiogenesis-related proteins: plasminogen activator inhibitor-1 (PAI-1), dipeptidyl peptidase-4 (DPPIV/CD26), and pigment epithelium-derived factor (PEDF), in Huh-7SR cells incubated with lenvatinib or DMSO for 24 h. Data are presented from three independent experiments. * *p* < 0.05, ** *p* < 0.01.

To determine whether lenvatinib affected the apoptosis and cell cycle in Huh-7SR cells, these cells were treated with lenvatinib, and FCM was performed. The Huh-7SR cells were treated with 10 μM lenvatinib or DMSO for 24 h, and the DMSO-treated cells were used as controls. The proportion of early apoptotic cells (lower right quadrant) among lenvatinib-treated cells was significantly higher than that among untreated cells (Figure 2A). Additionally, the expression of caspase-cleaved cytokeratin 18, cCK-18, a marker of apoptosis and necrosis, was upregulated in the lenvatinib-treated cells (Figure 2B). Furthermore, in terms of cell cycle progression, the cell population in the G0/G1 phase significantly increased, whereas cells in the S and G2/M phases decreased, suggesting that lenvatinib-treated cells were arrested in the G1 phase (Figure 2C). Invasion and wound healing assays showed that lenvatinib (10 μM) decreased invasion and migration ability after incubation for 24 h (Figure 2D,E). Further, in the Huh-7SR cells, lenvatinib affected the regulation of the expression of angiogenesis-related proteins: dipeptidyl peptidase-4 (DPPIV/CD26) [17,18,19] and pigment epithelium-derived factor (PEDF) [20] were upregulated, and plasminogen activator inhibitor-1 (PAI-1) [21] was downregulated, after incubation with 10 μM lenvatinib for 24 h (Figure 2F). 

### 2.3. Lenvatinib Induces Cell Cycle G1 Phase Arrest, Decreases Migration Ability, and Regulates the Expression of Angiogenesis-Related Proteins in Hep-3BSR Cells

**Figure 3:**
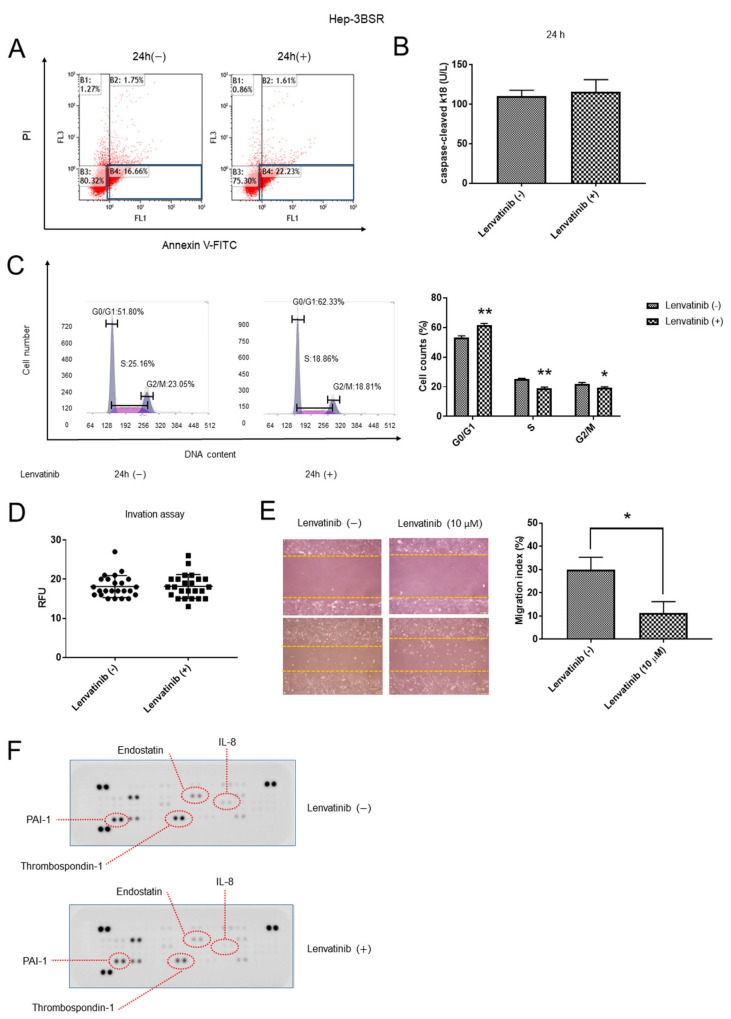
In Hep-3BSR cells, lenvatinib triggers cell cycle G1 phase arrest, reduces migration, and modulates the expression of proteins associated with angiogenesis. (**A**) Hep-3BSR cells were treated with 10 μM lenvatinib or dimethyl sulfoxide (DMSO) for 24 h, and the level of apoptosis was measured by staining with annexin V and propidium iodide (PI) using flow cytometry. Lenvatinib treatment did not significantly change the average proportion of early apoptotic cells in the Hep-3BSR population. Lower right square represents early apoptosis. (**B**) The expression of caspase-cleaved cytokeratin 18 (cCK-18) was determined using Enzyme Linked Immunosorbent Assay (ELISA). After 24 h of treatment with 10 μM lenvatinib, cCK-18 levels remained changed. (**C**) Hep-3BSR cells treated with 10 μM lenvatinib or DMSO were analyzed using flow cytometry to determine the number of cells in each phase of the cell cycle (left panel). Representative cell cycle histograms are presented (right panel). Lenvatinib blocked the cell cycle at the G0/G1 phase. (**D**) Invasion ability of lenvatinib-treated Hep-3BSR cells was not significantly changed. (**E**) Wound-healing assay comparing the motility of Huh-7SR cells treated with lenvatinib or DMSO. The wound-healing area was analyzed using ImageJ software. (**F**) Representative expression of angiogenesis-related proteins, endostatin, thrombospondin-1 (TSP-1), interleukin-8 (IL-8), and PAI-1, in Hep-3BSR cells incubated with lenvatinib or DMSO for 24 h. Data are presented from three independent experiments. * *p* < 0.05, ** *p* < 0.01.

The Hep-3BSR cells were treated with 10 μM lenvatinib or DMSO for 24 h, and the DMSO-treated cells were used as controls. No significant differences were found between the average proportion of early apoptotic cells in the lenvatinib-treated cells and the control (Figure 3A). Moreover, no changes were observed in the expression of cCK-18 between the lenvatinib-treated and untreated cells (Figure 3B). In cell cycle progression, the cell population in the G0/G1 phase significantly increased, whereas the cells in the S and G2/M phases decreased, suggesting that the lenvatinib-treated cells were arrested in the G1 phase (Figure 3C). The invasion assay suggested no difference between the control and treatment groups (Figure 3D). The wound healing assay indicated that 10 μM lenvatinib decreased migration ability after incubation for 24 h (Figure 3E). Additionally, the expression of angiogenesis-related proteins such as endostatin [22], thrombospondin-1 (TSP-1) [23,24], interleukin-8 (IL-8) [25], along with PAI-1, were downregulated after incubation with 10 μM lenvatinib for 24 h (Figure 3F). The expression of PAI-1 was downregulated in both the Huh-7SR and Hep-3BSR cells after lenvatinib treatment.

### 2.4. Lenvatinib Effects against Sorafenib-Resistance in HCC Cells May Be through the FGFR4-ERK Signaling Pathway

**Figure 4:**
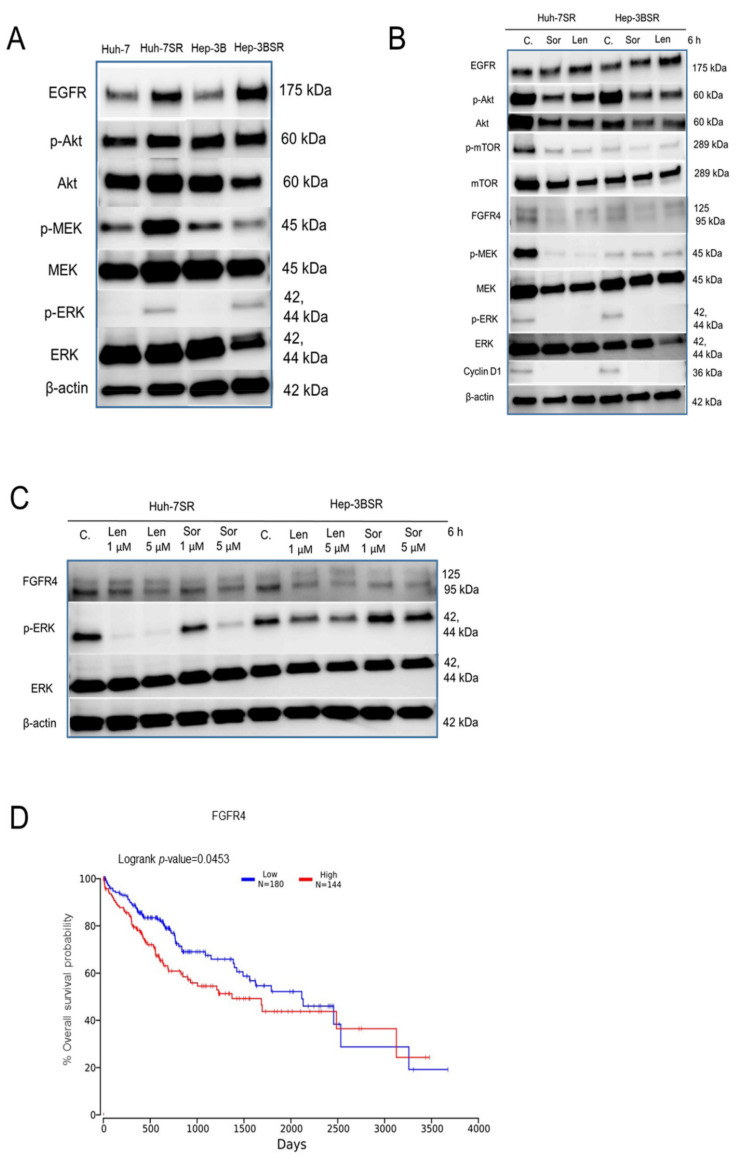
The underlying mechanisms of lenvatinib in sorafenib-resistant HCC cells may be through the FGFR4-ERK signaling pathway. (**A**) Proteins expressed in wild-type and sorafenib-resistant HCC cells. (**B**) Huh-7SR and Hep-3BSR cells were treated with 10 μM sorafenib or lenvatinib for 6 h. Protein expression was analyzed using Western blot. (**C**) Huh-7SR and Hep-3BSR cells were treated with 1 and 5 μM sorafenib or lenvatinib for 6 h, and the levels of FGFR4, p-ERK, and ERK were analyzed using Western blot. C.: control; Sor: sorafenib; Len: lenvatinib. (**D**) A lower FGFR4 level is associated with longer overall survival of HCC patients based on TCGA database analysis (http://www.oncolnc.org, accessed on 5 October 2021).

The expression levels of EGFR, p-Akt, and p-ERK were found to be upregulated in the sorafenib-resistant cells compared to the wild-type cells (Figure 4A). These results were similar to those reported in previous studies [6,7,8], showing that the overexpression of EGFR activated the Akt and ERK signaling pathway to increase HCC cell survival and proliferation, which, in turn, induced sorafenib resistance. The kinase inhibition profile for lenvatinib and sorafenib indicated that lenvatinib has an advantage in FGFR4 inhibition compared with sorafenib (Table S1). Previous studies have shown that Huh-7 and Hep-3B have high FGFR4 expression [26,27]. In the Huh-7SR and Hep-3BSR cells, 10 μM sorafenib or lenvatinib decreased the expression of p-Akt, Akt, FGFR4, p-ERK, and cyclinD1, whereas the levels of p-mTOR and mTOR remained unchanged in the Hep-3BSR cells. Meanwhile, lenvatinib increased the expression of EGFR (Figure 4B). The effects of lenvatinib in the two resistant cells were mainly through FGFR4-ERK signaling; however, the difference between 10 μM sorafenib and lenvatinib treatment was not significant. After decreasing the dosage, lenvatinib showed a superior inhibition of ERK compared with the same dosage of sorafenib (Figure 4C). In addition, the low expression of FGFR4 was associated with a longer overall survival probability based on the TCGA database analysis (Figure 4D).

### 2.5. Different Autophagic Responsiveness between Huh-7SR and Hep-3BSR Cells

**Figure 5:**
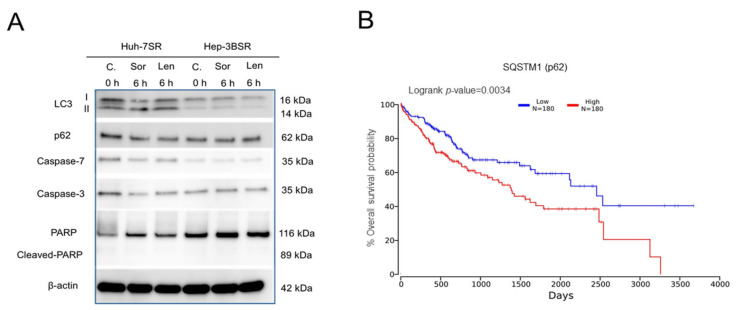
Autophagic response of sorafenib-resistant cells to sorafenib or lenvatinib. (**A**) Huh-7SR and Hep-3BSR cells were treated with 10 μM sorafenib or lenvatinib for 6 h. LC3, p62, caspase-7, caspase-3, and PARP expression were analyzed using Western blot. C.: control; Sor: sorafenib; Len: lenvatinib. (**B**) A lower p62 level is associated with longer overall survival of HCC patients based on TCGA database analysis (http://www.oncolnc.org, accessed on 5 October 2021).

Huh-7 and Hep-3B wild-type cells are both sensitive to lenvatinib (Figure S1). The IC_50_ data indicated that the Hep-3BSR cells showed partial cross-resistance to lenvatinib compared with the Huh-7SR cells. Moreover, lenvatinib inhibited Hep-3BSR cell proliferation mainly through cell cycle arrest, whereas in Huh-7SR, cell proliferation was inhibited through both apoptosis and cell cycle arrest. In addition, although mTOR is a key molecule in the regulation of autophagy, its activation remained changed after sorafenib or lenvatinib treatment in the Hep-3BSR cells. Autophagy plays a dual role in cancer development and is also associated with multidrug resistance in cancer cells [7,28]; thus, it was important to investigate if the autophagic responsiveness to sorafenib and lenvatinib is different between the two cell lines. In the Huh-7SR cells, 10 μM sorafenib or lenvatinib significantly increased the expression of microtubule-associated protein light chain 3-II (LC3-II); however, in the Hep-3BSR cells, the expression barely changed. Furthermore, in the Huh-7SR cells, while LC3-II levels increased, the expression of p62 decreased, indicating that 10 μM sorafenib or lenvatinib altered the autophagy in the Huh-7SR cells. Meanwhile, the levels of caspase-7 and caspase-3 were decreased, and the levels of PARP and cleaved-PARP were increased in the Huh-7SR cells, but not in the Hep-3BSR cells (Figure 5A). The Huh-7SR cells showed higher autophagic responsiveness to sorafenib and lenvatinib than the Hep-3BSR cells did. Lenvatinib induced high autophagic responsiveness that may induce autophagic cell death, and this may be one of the reasons that the Hep-3BSR cells showed partial cross-resistance to lenvatinib. Moreover, a lower p62 level is associated with the longer overall survival of HCC patients based on TCGA database analysis (Figure 5B).

### 2.6. Lenvatinib Affects microRNA Expression in Sorafenib-Resistant Cells

**Figure 6:**
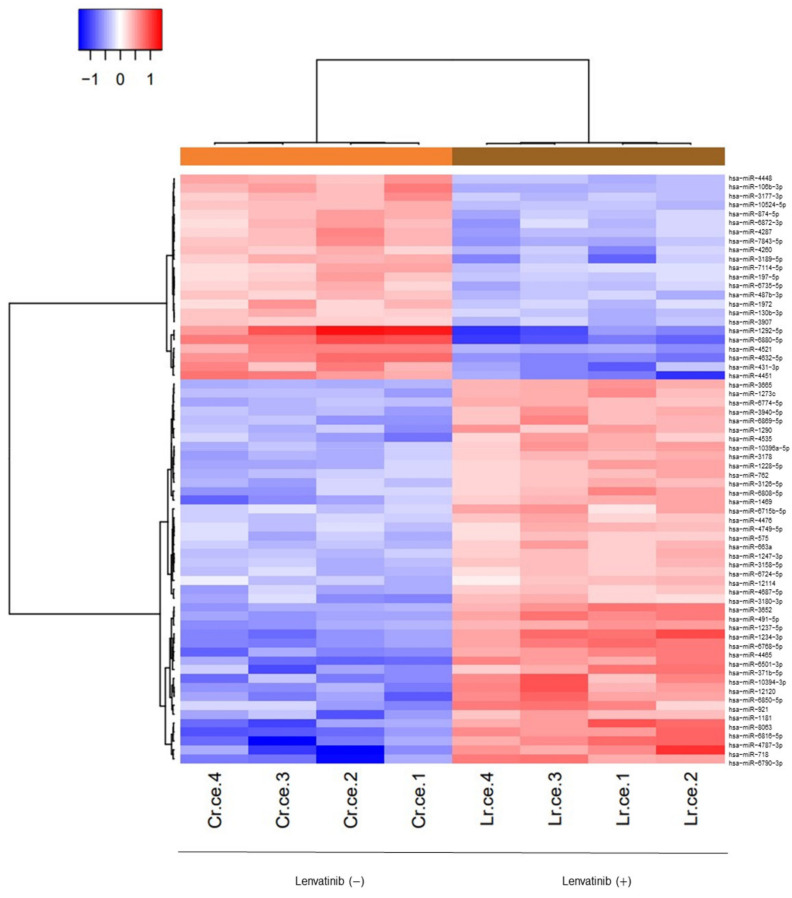
Lenvatinib affects miRNA expression. Hierarchical clustering of differentially expressed miRNAs from Huh-7SR cells incubated with 10 μM lenvatinib or DMSO for 24 h. Fold Change >1.5 or <0.67, *p* < 0.001.

A customized microarray platform was used to analyze the expression of 2555 miRNAs in the lenvatinib-treated or control Huh-7SR cells. Treatment with 10 μM lenvatinib for 24 h upregulated the expression (fold change > 1.5) of 43 miRNAs, such as miR-575, miR-663a, miR-491-5p, miR-4465, miR-371b-5p, and miR-718, and suppressed the expression (fold change < 0.67) of 23 miRNAs, such as miR-4448, miR-106b-3p, miR-197-5p, and miR-130b-3p (Table 1). Unsupervised hierarchical clustering analysis was conducted by calculating Pearson’s centered correlation coefficient, and the results indicated that the lenvatinib-treated Huh-7SR cells clustered together (Figure 6). Additionally, filtration (FDR < 0.001) indicated that lenvatinib upregulated the expression of 16 miRNAs and suppressed the expression of six miRNAs (Figure S2).

### 2.7. Altered miRNA in HCC and Normal Tissues and the Relationship with Overall Survival of HCC Patients Based on TCGA Database Analysis

**Figure 7:**
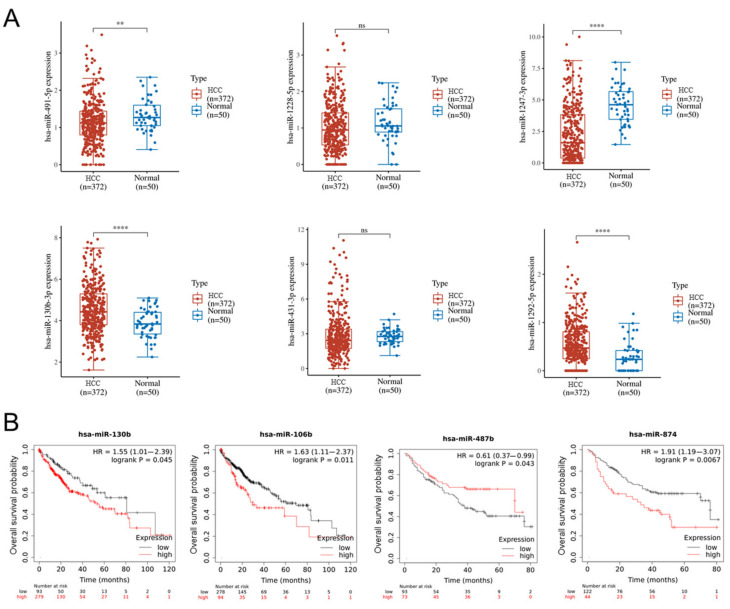
Altered microRNA expression and the relationship with overall survival in HCC. (**A**) Expression distribution of altered miRNAs after lenvatinib treatment in HCC and normal tissues based on TCGA database analysis, where different colors represent different groups. ** *p* < 0.01, **** *p* < 0.001, ns, no significant. (**B**) Lower levels of miR-130b, miR-106b, and miR-874, and higher levels of miR-487 are associated with longer overall survival of HCC patients (http://kmplot.com/analysis/, accessed on 5 October 2021).

Based on the altered miRNA expressions, 372 HCC tissues and 50 normal tissues from The Cancer Genome Atlas Liver Hepatocellular Carcinoma (TCGA-LIHC) database were analyzed. miR-130b-3p and miR-1292-5p are highly expressed, while the levels of miR-491-5p and miR-1247-3p are decreased in HCC tissues compared with normal tissues. No differences were found in the levels of miR-1228-5p and miR-431-3p between the HCC tissues and the normal tissues (Figure 7A). After lenvatinib treatment, miR-491-5p and miR-1247-3p were upregulated, while miR-130b-3p and miR-1292-5p were downregulated in the Huh-7SR cells. In addition, lower levels of miR-130b, miR-106b, and miR-874, and higher levels of miR-487, are associated with the longer overall survival of HCC patients (Figure 7B).

### 2.8. Lenvatinib Inhibits Huh-7 Sorafenib-Resistant Cell Proliferation In Vivo

**Figure 8:**
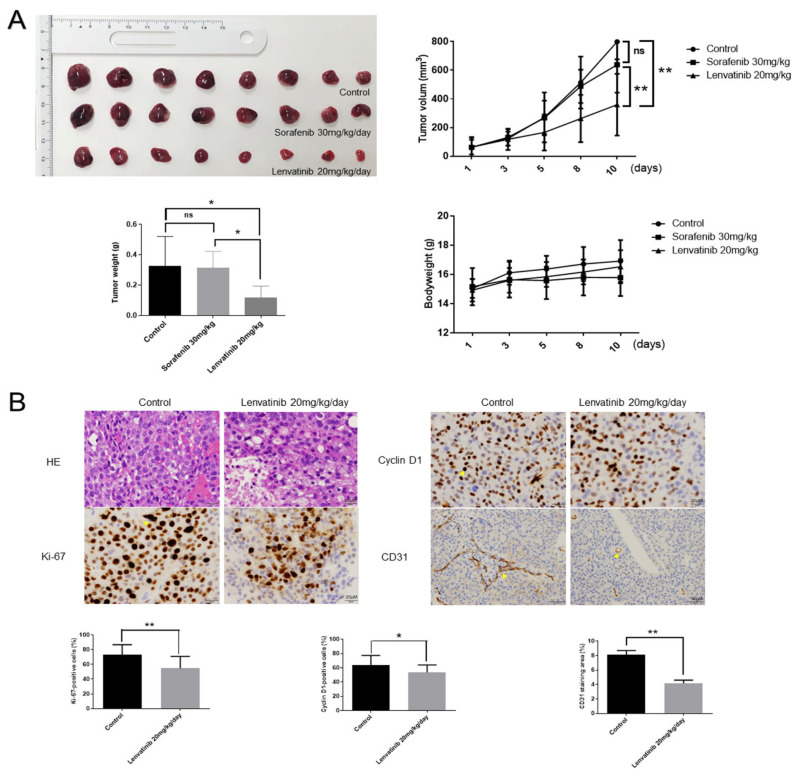
Lenvatinib inhibits Huh-7 sorafenib-resistant cell proliferation in vivo. (**A**) Lenvatinib 20 mg/kg/day (5 days/week) significantly inhibits tumor growth compared with control and sorafenib 30 mg/kg/day (5 days/week) (n = 8 per group). (**B**) Hematoxylin and eosin (H&E) staining and immunohistochemical staining of ki-67, cyclin D1 and CD31 proteins in the subcutaneous xenograft model. Ki-67-positive cells and cyclin D1-positive cells in the lenvatinib-treated groups were reduced in numbers compared with that in the control group. CD31 staining area in the lenvatinib-treated groups was decreased compared with that in the control group. Data are presented as mean ± standard deviation (SD). * *p* < 0.05, ** *p* < 0.01, ns, no significant.

Next, we examined the effect of lenvatinib in a nude mice xenograft model by injecting Huh-7SR cells. Tumor growth was significantly inhibited in the group treated with lenvatinib 20 mg/kg/day (5 days/week) compared with the sorafenib 30 mg/kg/day (5 days/week) and control groups (Figure 8A). Moreover, there was no difference in body weight among these three groups. Hematoxylin and eosin (H&E) staining and immunohistochemical staining of the ki-67, cyclin D1, and CD31 proteins in the subcutaneous xenograft model suggested that lenvatinib treatment decreased the levels of ki-67, cyclin D1, and the staining area of CD31, and suppressed cell proliferation and angiogenesis (Figure 8B).

## 3. Discussion

**Figure 9:**
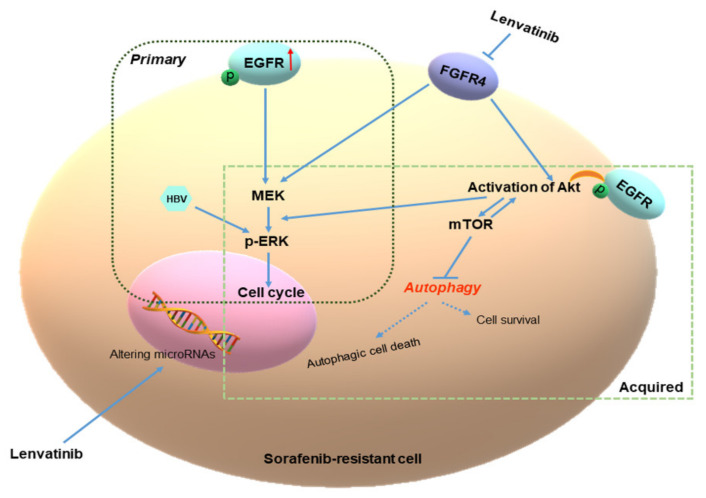
Proposed model for the underlying mechanism of lenvatinib in overcoming sorafenib resistance. Overexpression of EGFR leads to the activation of ERK and Akt signaling to induce sorafenib resistance and promote sorafenib-resistant cell proliferation. The underlying advantage of lenvatinib is the inhibition of FGFR4 compared with sorafenib. Huh-7SR and Hep-3BSR cells have high FGFR4 expression and the FGFR4-ERK signaling pathway is the major pathway to overcome increased EGFR-induced ERK activation. Compared with Huh-7SR cells, Hep-3BSR cells showed poor autophagic responsiveness to lenvatinib, which may contribute to their partial cross-resistance to lenvatinib.

The multi-kinase inhibitor, sorafenib, has been used as first-line therapy for patients with progressive unresectable HCC for a decade. However, resistance to sorafenib limits patient response and presents a major hurdle during HCC treatment. Additionally, data from the REFLECT trial indicated that lenvatinib was the first agent to achieve non-inferiority against sorafenib [13]. Therefore, in the present study, we evaluated the inhibition efficacy of lenvatinib in sorafenib-resistant HCC cells (Huh-7SR and Hep-3BSR cells). Key points include the following: (i) lenvatinib suppressed sorafenib-resistant HCC cell proliferation, mainly by inducing G1 cell cycle arrest; (ii) the underlying advantage of lenvatinib in overcoming sorafenib resistance may occur through the FGFR4-ERK signaling pathway; (iii) along with HBV DNA, poor autophagic responsiveness may be a contributing factor toward partial cross-resistance; and (iv) miRNA alterations may contribute to the inhibition of sorafenib-resistant HCC cell growth and angiogenesis (Figure 9).

The ERK signaling pathway plays a key role in anti-tumor effects and multi-kinase inhibitor resistance [28]. In both primary sorafenib-resistance and acquired resistance, the activation of ERK is major process that promotes cell proliferation. In addition, previous studies have revealed that HBx also activates the ERK signaling pathway in HCC [29]. The kinase inhibition profiles of lenvatinib and sorafenib indicated that lenvatinib is more effective at inhibiting FGFR4 (Table S1) [30,31]. Additionally, Huh-7SR and Hep-3BSR cells both expressed FGFR4 and EGFR. Further experiments showed that lenvatinib presented a better performance in ERK signaling inhibition compared with sorafenib. Thus, our findings suggest that lenvatinib overcomes sorafenib resistance mainly through the inhibition of the FGFR4-ERK signaling pathway. Previous studies also indicated that lenvatinib could strongly inhibit the activation of ERK, the downstream signaling molecules of FGFR4, compared with sorafenib and regorafenib [32], and high FGFR4 levels (positive immunohistochemistry >10% of tumor cells) were an independent predictor of a response to lenvatinib [33]. Moreover, lenvatinib enhanced the antitumor immune response of anti-programmed cell death-1 (PD-1) in HCC by blocking FGFR4 [34]. However, lenvatinib alone could inhibit HCC cancer stem-like cells through FGFR1-3 signaling, but not FGFR4 signaling [35]. Interestingly, lenvatinib could also increase the expression of EGFR, as previously reported [36,37]. The activation of EGFR may contribute to sorafenib or lenvatinib resistance. The combination therapy of lenvatinib and gefitinib (an EGFR inhibitor) may be an option for the approximately 50% of advanced HCC patients with high EGFR expression [37]. Combination therapy or new kinase inhibitors (such as ERK inhibitors) may represent a promising strategy for sorafenib-resistant HCC patients.

Moreover, we found that Huh-7SR cells showed higher autophagic responsiveness to sorafenib and lenvatinib than Hep-3BSR cells, and this may contribute to Hep-3BSR’s partial cross-resistance to lenvatinib. Autophagy plays neutral, tumor-suppressive, or tumor-promoting roles in cancer development, and is also associated with apoptosis and multidrug-resistance [38]. Previous studies demonstrated that the autophagic responsiveness to sorafenib is distinct between Hep3B and Huh7 wild-type cells and the sensitivity to sorafenib is also different [39], where sorafenib-induced autophagy improves the death rate of HCC cells [40]. Similarly, in sorafenib-resistant cells, the different autophagic responsiveness to lenvatinib may also be associated with altered sensitivity to lenvatinib.

miRNAs are a class of endogenous, small, noncoding-RNA molecules that regulate aspects of the post-transcriptional modulation of gene expression, such as cell proliferation, differentiation, metabolism, and cell death [41,42]. Additionally, several reports have shown that miRNAs can regulate the sensitivity of HCC cells to multi-kinase inhibitor drugs, such as sorafenib, by modifying diverse molecular processes [43,44]. In this study, we reported altered cell miRNA expression in Huh-7SR cells after treatment with lenvatinib. A total of 43 miRNAs were upregulated, while 23 miRNAs were downregulated. Further data set analysis from TCGA indicated that miR-130b-3p and miR-1292-5p were highly expressed, while the levels of miR-491-5p and miR-1247-3p were decreased in HCC tissues compared with normal tissues. In addition, miRNA expression was reversed after lenvatinib treatment. In many cases, miRNAs can have oncogenic effects or act as suppressors in different cancers, and some may also have a dual function. Liao et al. indicated that miR-130b-3p was upregulated in HCC and was correlated with a poor prognosis. Overexpressed miR-130b-3p was found to enhance the angiogenesis capacity of HCC cells [45]. Decreased miR-491-5p and highly pyruvate kinase M2 (PKM2) expression were associated with unfavorable clinical features and the poor prognosis of HCC patients [46]. In addition, overexpressed miR-491-5p inhibited HCC cell proliferation and migration by targeting SEC61 translocon alpha 1 subunit1 (SEC61A) [47]. The inhibition effect of lenvatinib in sorafenib-resistant HCC cells occurred partially through miRNA regulation. However, due to the scarcity of information on some of the miRNAs, further studies are needed on their function.

Only a few clinical studies have been conducted on second-line or further-line treatment of lenvatinib. Jefremow et al. suggested that in seven patients with a later line lenvatinib treatment, partial remission (PR) was shown in four of the seven patients, stable disease (SD) in two of the seven, and mixed response with overall tolerable safety in one of the seven [48]. Chen et al. enrolled 40 patients who received lenvatinib after sorafenib. The median overall survival (OS) was 9.8 months, and the objective response rate was 27.5%; moreover, the clinical outcomes of lenvatinib treatment in later lines were similar [15]. However, a report by Tomonari et al. indicated that the objective response rate was 33.3% in the second line, and 20.0% in the third line. Additionally, sorafenib-resistant HCC cells show partial cross-resistance to lenvatinib by the decreased response to FGFR signaling pathways compared with wild-type cells [16] (Table 2). Although the number of patients was one of the limitations of the study, an effective tendency was shown.

This study has the following several limitations: (i) We only used two sorafenib-resistant HCC cell lines; (ii) expanded clinical studies are needed, including in sorafenib-resistant HCC patients with HBV infection, sorafenib-resistant HCC patients with high or low FGFR4 expression, and an investigation of a promising strategy, such as combination therapy with lenvatinib. Hence, we plan to perform further experiments to investigate the above limitations in our future research on this topic.

In conclusion, our study shows that (i) the advantage of lenvatinib in overcoming sorafenib-resistance may be through the FGFR4-ERK signaling pathway; (ii) HBV DNA and poor autophagic responsiveness may be the reasons for partial cross-resistance; (iii) miRNA alterations may contribute to inhibiting sorafenib-resistant HCC cell growth and angiogenesis. The present study and previous clinical data (Table 2) provide evidence that lenvatinib may be a suitable second-line therapy for unresectable HCC patients who express FGFR4 and are sorafenib resistant. Combination therapy could be a promising way to expand the efficiency of first-line multi-kinase inhibitors and our study is one of the initial investigations of this topic. Drug resistance in HCC patients is a huge barrier to overcome. Attenuating first-line treatment resistance and expanding the efficiency of second-line therapy could potentially prolong the survival time of patients. Thus, further studies and clinical trials are needed.

## 4. Materials and Methods

### 4.1. Chemicals

Sorafenib (Nexavar^®^) was obtained from Bayer Pharmaceutical Corporation (West Haven, CT, USA) and lenvatinib from Chem Scene (Monmouth Junction, NJ, USA). Matrigel^®^ Matrix (356230) was purchased from Corning Inc. (Lowell, MA, USA).

### 4.2. Cell Lines and Cell Culture

Human HCC cell lines, Huh-7 and Hep-3B, were obtained from the Japanese Research Resources Bank (Tokyo, Japan). Huh-7 cells were maintained in Dulbecco’s Modified Eagle’s Medium (DMEM; Gibco-Invitrogen, Carlsbad, CA, USA) supplemented with 10% fetal bovine serum (FBS; Wako, Tokyo, Japan) and penicillin/streptomycin (100 mg/L; Invitrogen, Tokyo, Japan). Hep-3B cells were cultured in Modified Eagle’s Medium (MEM; Gibco-Invitrogen) supplemented with 10% FBS and penicillin/streptomycin. To establish sorafenib-resistant HCC cell lines, Huh-7 and Hep-3B cells were cultured with increasing doses of sorafenib, from 1 to 6 μM, for six months. Cells were grown in a humidified incubator at 5% CO_2_ and 37 °C. Huh-7 and Hep-3B cell lines as well as Huh-7 sorafenib-resistant (Huh-7SR) and Hep-3BSR sublines were authenticated using short tandem repeat (STR) profiling (BEX Co., Ltd., Tokyo, Japan).

### 4.3. Cell Viability Assay

Cell viability assays were performed using the Cell Counting Kit-8 (Dojindo Laboratories, Kumamoto, Japan) according to the manufacturer’s instructions. Briefly, the cells were seeded in 96-well plates at a concentration of 5000 cells/100 µL/well and incubated in a normal growth medium for 24 h. Subsequently, the cells were grown for an additional 24, 48, 72, or 96 h with sorafenib (0.3, 1, 3, 10, and 30 μM), lenvatinib (0.3, 1, 3, 10, and 30 μM), or dimethyl sulfoxide (DMSO). The medium was replaced with 100 µL of fresh medium containing 10% CCK-8 reagent, and the cells were incubated at 37 °C for 3 h. The absorbance was measured at 450 nm using a multi-grating microplate reader SH-9000Lab (CORONA Electric Co., Ltd., Ibaraki, Japan). The experiments were repeated three times.

### 4.4. Three-Dimensional (3D) Tumor Spheroid Assay (3D Culture)

Multicellular spheroids were generated using the liquid overlay technique (Figure S3) [49].

### 4.5. Flow Cytometry Analysis of Cell Cycle

Cell cycle progression was evaluated using a Cell Cycle Phase Determination Kit (Cayman Chemical Company, Ann Arbor, MI, USA). Cells (1.0 × 10^6^ cells/100-mm dish) were treated with 10 μM lenvatinib or DMSO for 24 h. Cells were trypsinized and resuspended in phosphate-buffered saline (PBS) at a density of 10^6^ cells/mL. Approximately 1.0 × 10^6^ cells were stained in 100 µL of PBS with 10 µL of RNase A (250 µg/mL) and 10 µL of propidium iodide (PI) stain (100 µg/mL) and incubated at room temperature in the dark for 30 min. Flow cytometry (FCM) was performed to compare the proportion of lenvatinib-treated and control cells in each phase of the cell cycle. FCM was performed using a Cytomics FC 500 flow cytometer (Beckman Coulter, Brea, CA, USA) equipped with an argon laser (488 nm), and the percentage of cells was analyzed using the Kaluza software version v2.1 (Beckman Coulter). The experiments were repeated three times.

### 4.6. Apoptosis Analysis

Lenvatinib-mediated apoptosis was analyzed using an FCM and Annexin V-FITC Early Apoptosis Detection kit (Cell Signaling Technology, Beverly, MA, USA). Cells (1.0 × 10^6^ cells/100-mm dish) were treated with 10 μM lenvatinib or DMSO for 24 h. Apoptotic and necrotic cells were analyzed by double staining with FITC-conjugated annexin V and PI according to the manufacturer’s instructions. FCM was conducted using a Cytomics FC 500 flow cytometer equipped with an argon laser (488 nm) to compare the proportion of apoptotic cells in the lenvatinib-treated and control groups, and data were analyzed using the Kaluza software version v2.1. The experiments were repeated three times.

### 4.7. Apoptosis Analysis by Enzyme Linked Immunosorbent Assay (ELISA)

ELISA was performed to analyze the levels of caspase-cleaved cytokeratin 18 (cCK18) using the M30-Apoptosense ELISA kit (Peviva Ab, Bromma, Sweden). Cells (5000 cells/well) were seeded in 96-well plates and treated with 10 μM lenvatinib or DMSO for 24 h. Subsequently, the cells were lysed in polyoxyethylene octyl phenyl ether (Wako) and analyzed according to the manufacturer’s instructions. The experiments were repeated three times.

### 4.8. Antibody Arrays to Analyze Angiogenesis-Related Proteins

Cells (1.0 × 10^6^ cells/100-mm dish) were treated with 10 μM lenvatinib or DMSO for 24 h at 37 °C and lysed with a protease inhibitor cocktail, PRO-PREP complete protease inhibitor mixture (iNtRON Biotechnology, Seongnam, Korea). The Human Angiogenesis Array Kit (R&D Systems) was used to analyze the angiogenesis-related proteins in the lenvatinib-treated and control cells according to the manufacturer’s protocol. Each array was repeated three times to validate the results.

### 4.9. Invasion Assay

For invasion assays, 1 × 10^4^ cells were cultured in 100 μL of serum-free DMEM or with 10 μM lenvatinib, placed into the upper membrane chamber (Cell Biolabs, San Diego, CA, USA.) and precoated with extracellular matrix (ECM) proteins. The lower compartment was filled with 150 μL of DMEM containing 10% FBS. After incubation for 24 h in an atmosphere of 5% CO_2_ at 37 °C, the cells in the upper compartment were removed, and the invested cells in the lower compartment were lysed. Fluorescence was read using an SH-9000Lab plate reader (CORONA) at 480 nm/520 nm.

### 4.10. Wound Healing Assay

Cells were seeded in 6-well plates and incubated in DMEM containing 10% FBS until they reached subconfluence. Scratches were introduced in the cell monolayer using a plastic pipette tip. After washing with PBS, 1.5% FBS media containing 10 μM lenvatinib or DMSO was added. The scratched area was photographed using a light microscope after 24 h.

### 4.11. miRNA Microarray

Total RNA of cells was extracted using the miRNeasy Mini Kit (QIAGEN) according to the manufacturer’s instructions. After confirming the purity and quantity of each sample using an Agilent 2100 Bioanalyzer (Agilent Technologies, Santa Clara, CA, USA) and an RNA 6000 Nano kit (Agilent Technologies), respectively, the samples were labeled using a miRCURY Hy3 Power Labeling kit (Exiqon A/S, Vedbaek, Denmark) and hybridized to a human miRNA Oligo Chip (v.21; Toray Industries, Inc., Tokyo, Japan). Chips were scanned using a 3D-Gene Scanner 3000 (Toray Industries). The 3D-Gene extraction software version 1.2 (Toray Industries) was used to calculate the raw signal intensity of the images. The raw data were analyzed using GeneSpring GX 10.0 software (Agilent Technologies) to assess the differences in miRNA expression between the samples. Global normalization was performed on raw data obtained above the background level. Differentially expressed miRNAs were determined using Welch’s *t*-test.

### 4.12. Colony Formation Assay

Cells were trypsinized for 3 min and resuspended at a density of 1 × 10^3^/mL. Five hundred microliters were seeded into 6-well plates, and 1.5 mL of DMEM containing 10% FBS and 1 μΜ lenvatinib or DMSO was added to each well. The plates were incubated at 37 °C in 5% CO_2_, and the medium was changed every three days until conspicuous colonies were observed. Colonies were fixed and stained with 0.1% crystal violet at room temperature for 5 min, and positive colony formation (>50 cells/colony) was evaluated by counting the number of colonies.

### 4.13. Data Set Analysis

All patients of LIHC were retrieved from the TCGA data portal (https://portal.gdc.cancer.gov/, accessed on 3 September 2021). The full clinical dataset was downloaded (up to 3 September 2021) and this study meets the publication guidelines provided by TCGA (http://cancergenome.nih.gov/publications/publicationguidelines, accessed on 3 September 2021) [50,51]. OncoLnc (http://www.oncolnc.org, accessed on 5 October 2021) [52] and KM plotter (http://kmplot.com/analysis, accessed on 5 October 2021) [53] were used for survival analysis.

### 4.14. Western Blot

The cells were lysed with a PRO-PREP complete protease inhibitor mixture (iNtRON Biotechnology, Korea) and collected supernatants. Protein concentration was measured using a NanoDrop 2000 spectrofluorometer (Thermo Fisher Scientific, Inc., Waltham, MA, USA). Briefly, protein aliquots (10 µg) were separated on precast protein gels (4–20% Mini-PROTEAN TGX Gels; Bio-Rad, Hercules, CA, USA) and transferred onto nitrocellulose membranes. The membranes were blocked with 2% skimmed milk (GE Healthcare) in TBST with 0.1% Tween 20 (cat. No. T9142, Takara Bio Inc., Kusatsu, Shiga, Japan) for 30 min and were then incubated overnight at 4 °C with the following primary antibodies: anti-ß-Actin (#66009-1-lg, Proteintech, dilution 1:5000); anti-LC3A/B (#12741, CST, dilution 1:1000); SQSTM1/p62 (#5114, CST, dilution 1:1000); Caspase-3 (#14220, CST, dilution 1:1000); PARP (#9542, CST, dilution 1:1000); Caspase-7 (#12827, CST, dilution 1:1000); EGFR (#PAI-1110, Thermo Fisher Scientific, dilution 1:1000); p-Akt (ser473) (#4060, CST, dilution 1:2000); Akt (#4685, CST, dilution 1:1000); p-mTOR (SER2448) (#5536, CST, dilution 1:1000); mTOR (#2983, CST, dilution 1:1000); cyclinD1 (#2978, CST, dilution 1:1000); p-ERK1/2 (#4370, CST, dilution 1:1000); ERK1/2 (#4695, CST, dilution 1:1000); p-MEK1/2 (#9154, CST, dilution 1:1000); MEK1/2 (#8727, CST, dilution 1:1000); anti-ß-Actin (#4967, CST, dilution 1:1000); FGFR4 (#8562, CST, dilution 1:1000) in 5% serum (cat. No. 9048-46-8, FUJIFILM Wako, Osaka, Japan). After washing with TBST, the membranes were incubated for 1 h with corresponding horseradish peroxidase (HRP)-conjugated secondary antibodies: anti-mouse (#7076, CST, dilution 1:2000) or anti-rabbit (#7074, CST, dilution 1:2000). The signal was visualized using a chemiluminescent (ECL) kit (cat. No. 45-000-999; Cytiva) and imaged using ImageQuant LAS 4010 (GE Healthcare).

### 4.15. Xenograft Model Analysis

The animal study was approved (approval No. 20627, 20640 and 21681) by, and was conducted in accordance with the guidelines set by, the Committee on Experimental Animals of the Kagawa University. Female athymic mice (BALB/c-nu/nu; 5 weeks old; 15–17 g) were purchased from Japan SLC (Shizuoka, Japan). The mice were maintained under specific pathogen-free conditions using a laminar airflow rack and had continuous free access to sterilized (γ-irradiated) food (CL-2; CLEA Japan, Inc., Tokyo, Japan) and autoclaved water. Mice were subcutaneously inoculated with 5 × 10^6^ Huh-7SR cells in the right flank. When the xenografts were palpable with an approximate diameter of 5 mm, we randomly assigned the animals to three groups of eight mice each. These groups were orally administered 20 mg/kg/day lenvatinib, 30 mg/kg/day sorafenib, or vehicle (DMSO and saline) for five days per week. Tumor volume (mm^3^) was calculated as tumor length (mm) × tumor width (mm)^2^/2. All animals were sacrificed on day 10 of treatment.

### 4.16. Immunohistochemistry

Specimens were fixed overnight in 10% formalin, embedded in paraffin, and cut into sections 5 μm thick. Briefly, slides were deparaffinized in xylene, rehydrated through a graded alcohol series, and rinsed in PBS. Depending on the protein target to be revealed, antigen retrieval was achieved by boiling the sections in either 10 mM sodium citrate buffer (pH 6.0) or 1 mM ethylenediaminetetraacetic acid (EDTA; pH 8.5) buffer for 10 min, followed by a 20-min cool down at room temperature. After a blocking step using 5% goat serum and an Avidin-Biotin Blocking Kit (Vector Laboratories, Burlingame, CA, USA), the slides were incubated with specific primary antibodies (cyclin D1, ki-67, CD31) overnight at 4 °C. To quench the endogenous peroxidase activity, slides were incubated for 10 min with 3% hydrogen peroxide, and subsequently, the biotin-conjugated secondary antibody was applied at a 1:500 dilution for 30 min at room temperature. Immunoreactivity was visualized using the Vectastain Elite ABC kit (Vector Laboratories) and 3,3-diaminobenzidine or Vector NovaRed (Vector Laboratories) as the chromogen. Finally, the slides were counterstained with hematoxylin.

### 4.17. Statistical Analysis

GraphPad Prism software version 6.0 (GraphPad Software, San Diego, CA, USA) was used for all analyses. Unpaired Student’s *t*-test and Wilcox test were used to determine statistical significance between different groups. Two-way analysis of variance (ANOVA) or mixed ANOVA was performed to test the comparisons and corrected using Tukey’s post hoc test. One-way ANOVA was performed before the Tukey’s post hoc test to test the comparisons. Values were considered statistically significant at a *p*-value < 0.05.

## Figures and Tables

**Table 1:** Statistical results and chromosomal locations of miRNAs that exhibited a fold change (FC) > 1.5, FC < 0.67, or *p* < 0.001 in HCC Huh-7SR cells treated with lenvatinib when compared with untreated cells.

miRNA	*p*-Value	Fold Change (Treated/Untreated)	FDR	Chromosomal Location
Upregulated				
has-miR-718	0.000823533	2.93	0.009177565	Xq28
hsa-miR-4787-3p	0.000378539	2.82	0.005427912	3p21.2
hsa-miR-6816-5p	1.73835 × 10^−5^	2.69	0.000977428	22q11.21
hsa-miR-6790-3p	0.000369116	2.64	0.005427912	19p13.3
hsa-miR-1234-3p	2.9809 × 10^−5^	2.59	0.001292429	8q24.3
hsa-miR-8063	0.000246669	2.56	0.004141378	15q14
hsa-miR-6768-5p	2.43923 × 10^−6^	2.48	0.000450491	16p13.3
hsa-miR-6501-3p	1.236 × 10^−5^	2.47	0.000867423	21q22.11
hsa-miR-10394-3p	0.00019664	2.40	0.003800681	19q13.43
hsa-miR-6850-5p	6.5404 × 10^−5^	2.36	0.002074481	8q24.3
hsa-miR-12120	5.15411 × 10^-5^	2.35	0.001850097	Yq11.221
hsa-miR-4465	1.65012 × 10^−5^	2.35	0.000971998	6q24.1
hsa-miR-491-5p	1.25706 × 10^−6^	2.22	0.000388745	9p21.3
hsa-miR-371b-5p	0.000874032	2.20	0.009484016	19q13.42
hsa-miR-3652	1.24497 × 10^−5^	2.15	0.000867423	12q23.3
hsa-miR-921	0.000571511	2.04	0.007188459	1q24.1
hsa-miR-1237-5p	2.91345 × 10^−6^	2.03	0.000450491	11q13.1
hsa-miR-1469	0.000239265	1.95	0.004141378	15q26.2
hsa-miR-1181	0.000576271	1.92	0.007188459	19p13.2
hsa-miR-6869-5p	7.72727 × 10^−5^	1.88	0.00227243	20p13
hsa-miR-3665	5.65874 × 10^−7^	1.85	0.000349993	13q22.3
hsa-miR-1290	0.000146648	1.85	0.003194727	1p36.13
hsa-miR-1273c	3.34339 × 10^−5^	1.84	0.001292429	6q25.2
hsa-miR-4535	0.000456305	1.81	0.00613532	22q13.32
hsa-miR-10396a-5p	6.04782 × 10^−5^	1.791	0.001968724	21p11.2
hsa-miR-3940-5p	2.45816 × 10^-5^	1.78	0.001216297	19p13.3
hsa-miR-6808-5p	0.000904459	1.76	0.009562526	1p36.33
hsa-miR-1228-5p	0.000103027	1.76	0.002591924	12q13.3
hsa-miR-6774-5p	6.90461 × 10^−6^	1.74	0.000731907	16q24.1
hsa-miR-3180-3p	0.000507427	1.74	0.006677529	16p13.11
hsa-miR-3178	2.33963 × 10^−5^	1.73	0.001205886	16p13.3
hsa-miR-3126-5p	7.5417 × 10^−5^	1.69	0.00227243	2p13.3
hsa-miR-4687-5p	3.28134 × 10^−5^	1.67	0.001292429	11p15.4
hsa-miR-663a	8.50531 × 10^−5^	1.66	0.002355633	20p11.1
hsa-miR-3158-5p	6.85365 × 10^−6^	1.63	0.000731907	10q24.32
hsa-miR-762	0.000107378	1.62	0.00260445	16p11.2
hsa-miR-6715b-5p	0.00066953	1.62	0.007813291	10q25.2
hsa-miR-1247-3p	1.33234 × 10^−5^	1.60	0.000867423	14q32.31
hsa-miR-4476	4.84072 × 10^−5^	1.58	0.001814535	9p13.2
hsa-miR-4749-5p	8.89695 × 10^−5^	1.56	0.002355633	19q13.33
hsa-miR-575	0.00018907	1.56	0.003712369	4q21.22
hsa-miR-6724-5p	8.70242 × 10^−5^	1.55	0.002355633	21p11.2
hsa-miR-12114	0.000812774	1.51	0.009140012	22q13.33
Downregulated				
hsa-miR-7114-5p	0.000857372	0.65	0.009469368	9q34.3
hsa-miR-197-5p	0.000179571	0.65	0.003697546	1p13.3
hsa-miR-3907	1.10865 × 10^−5^	0.65	0.000867423	7q36.1
hsa-miR-1972	0.000596743	0.63	0.00723697	16p13.11
hsa-miR-6735-5p	9.42138 × 10^−5^	0.63	0.002427969	1p34.2
hsa-miR-130b-3p	7.89931E-05	0.63	0.00227243	22q11.21
hsa-miR-487b-3p	2.90019 × 10^−5^	0.63	0.001292429	14q32.31
hsa-miR-6872-3p	0.000677028	0.60	0.007826946	3p21.31
hsa-miR-10524-5p	4.7563 × 10^−7^	0.60	0.000349993	6q14.1
hsa-miR-4260	0.000425744	0.60	0.00587887	1q32.2
hsa-miR-3177-3p	8.95026 × 10^−5^	0.60	0.002355633	16p13.3
hsa-miR-874-5p	0.000111442	0.59	0.002651026	5q31.2
hsa-miR-4287	0.000214498	0.56	0.003919721	8p21.1
hsa-miR-4448	1.41712 × 10^−5^	0.56	0.000876488	3q27.1
hsa-miR-7843-5p	5.23471 × 10^−5^	0.54	0.001850097	14q24.2
hsa-miR-3189-5p	0.000538484	0.54	0.00693859	19p13.11
hsa-miR-106b-3p	3.19133 × 10^−5^	0.53	0.001292429	7q22.1
hsa-miR-4521	7.10015 × 10^−6^	0.47	0.000731907	17p13.1
hsa-miR-431-3p	0.00037795	0.46	0.005427912	14q32.2
hsa-miR-4451	0.000147211	0.40	0.003194727	4q21.23
hsa-miR-4632-5p	9.73972 × 10^−7^	0.39	0.000388745	1p36.22
hsa-miR-6880-5p	2.78965 × 10^−6^	0.31	0.000450491	12q24.31
hsa-miR-1292-5p	0.000166801	0.29	0.003557465	20p13

**Table 2:** Lenvatinib as second-line or further-line treatment after sorafenib.

Author	Total Patients	Treatment Line		Complete Remission	Partial Remission	Stable Disease	Mixed Response	Progression
Jefremow A et al. [48]	7	Second line	2	0	1	1	0	0
		Third line	3	0	2	0	1	0
		Fourth line	2	0	1	1	0	0
Chen YY et al. [15]	40	Second line	20	1	5	7	0	7
		Third line	10	0	4	4	0	2
		Fourth line	10	0	1	5	0	4
Tomonari T et al. [16]	19	Second line	9	1	2	5	0	1
		Third line	10	0	2	8	0	0
	66			2	18	31	1	14

## Data Availability

All data supporting the conclusions of the present study have been documented in this article.

## Supplementary Materials

The following are available online at https://www.mdpi.com/article/10.3390/ijms222313071/s1.
