# Supplementary material for: Evaluating the Effect of Lenvatinib on Sorafenib-Resistant Hepatocellular Carcinoma Cells
Source: Int J Mol Sci. 2021 Dec 2;22(23):13071. doi: 10.3390/ijms222313071 (PMC8657692; doi:10.3390/ijms222313071)
Supplement: Supplementary file 1 [file ijms-22-13071-s001.zip › Supplement_data.pdf]

Supplementary Materials: Evaluating the Effect of Lenvatinib on Sorafenib-Resistant Hepatocellular Carcinoma Cells.

Tingting Shi, Hisakazu Iwama, Koji Fujita, Hideki Kobara, Noriko Nishiyama, Shintaro Fujihara, Yasuhiro Goda, Hirohito Yoneyama, Asahiro Morishita, Joji Tani, Mari Yamada, Mai Nakahara, Kei Takuma, and Tsutomu Masaki

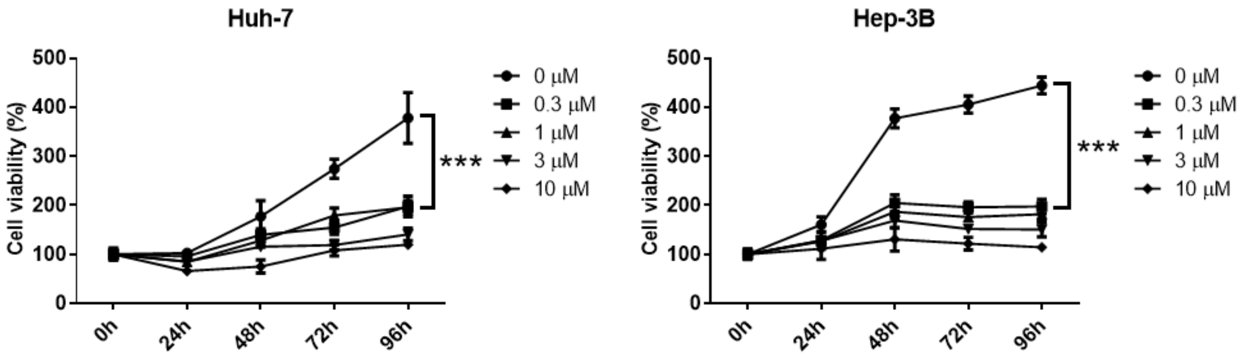

Figure S1. Lenvatinib inhibits the proliferation of wild-type human hepatocellular carcinoma (HCC) cells. Anti-proliferative effect of lenvatinib measured by cell viability assay: Huh-7 and Hep-3B cells were treated for 96 h with the indicated concentrations of lenvatinib or DMSO. The relative cell number was normalized with the control, *** $p < 0.001$.

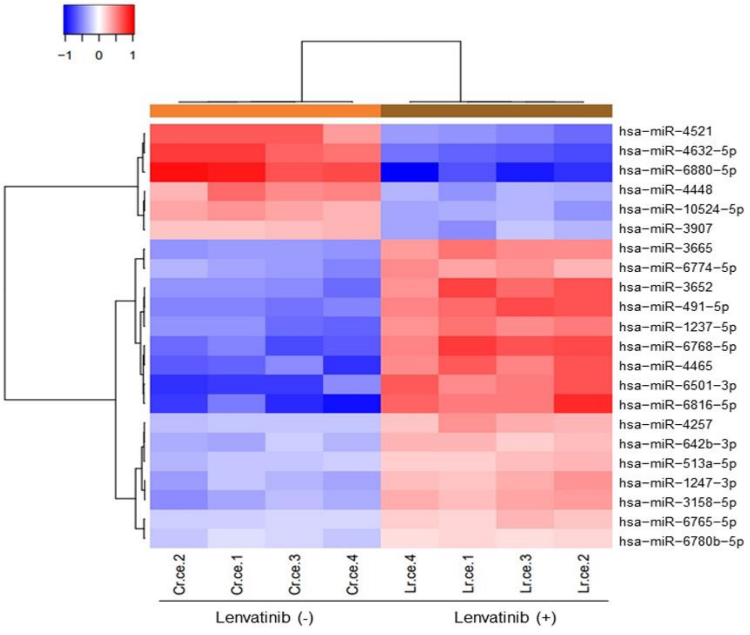

Figure S2. Lenvatinib affects microRNA expression in Huh-7SR cells. Hierarchical clustering of differentially expressed miRNAs from Huh-7SR cells incubated with 10 μM lenvatinib or DMSO for 24 h. Fold Change >1.5 or <0.67, FDR<0.001.

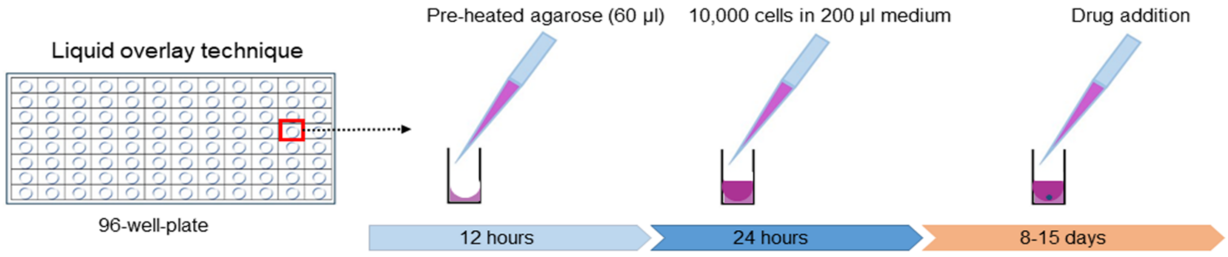

Figure S3. Schematic diagram of three-dimensional tumor spheroid assay by liquid overlay technique.

Table S1. Kinase inhibition profile.

TKR	Lenvatinib IC50 (μM)	Sorafenib IC50 (μM)
FGFR1	0.061	0.34
FGFR2	0.027	0.15
FGFR3	0.052	0.34
FGFR4	0.043	3.4
VEGFR1	0.0047	0.021
VEGFR2	0.003	0.021
VEGFR3	0.0023	0.016
RET	0.0064	0.015
